# Suberoylanilide hydroxamic acid-induced specific epigenetic regulation controls Leptin-induced proliferation of breast cancer cell lines

**DOI:** 10.18632/oncotarget.13764

**Published:** 2016-12-01

**Authors:** Xiuyan Feng, Han Han, Dan Zou, Jiaming Zhou, Weiqiang Zhou

**Affiliations:** ^1^ The Second Affiliated Hospital of Shenyang Medical College, Heping District, Shenyang City, Liaoning Province 110002, P. R. China; ^2^ Key Laboratory of Environmental Pollution and Microecology of Liaoning Province, Shenyang Medical College, Huanggu District, Shenyang City, Liaoning Province 110034, P. R. China; ^3^ Northeast Yucai Foreign Language School, Hunnan New District, Shenyang City, Liaoning Province 110179, P. R. China

**Keywords:** suberoylanilide hydroxamic acid, histone acetylation, breast cancer

## Abstract

Breast cancer is one of the most common malignancies among women in the world, investigating the characteristics and special transduction pathways is important for better understanding breast development and tumorigenesis. Leptin, a peptide hormone secreted from white adipocytes, may be an independent risk factor for breast cancer.

Here, we treated suberoylanilide hydroxamic acid (SAHA) on Leptin-induced cell proliferation and invasion in the estrogen-receptor-positive breast cancer cell line MCF-7 and triple-negative breast cancer cell line MDA-MB-231. Low concentrations of Leptin (0.625 nM) significantly stimulated breast cancer cell growth, enhanced cell viability, minimized apoptosis, and increased cell cycle transition. In contrast, SAHA (5 μM) treatment had reverse effects. Wound healing assay showed that, in MCF-7 and MDA-MB-231 cell line, cell migrating stimulated by Leptin was significantly repressed with SAHA treatment. Moreover, cell cycle real-time PCR array and proteome profiler antibody array confirmed that Leptin and SAHA treatment significantly changed the expressions of factors associated with cell cycle regulation and apoptosis including p53 and p21^WAF1/CIP1^.

In DNA-ChIP analysis, we found that acetylation levels binding with p21^WAF1/CIP1^ promoters are regulated in a manner specific to histone type, lysine residue and selective promoter regions. SAHA significantly up-regulated the acetylation levels of AcH3-k14 and AcH3-k27 in MCF-7 cells, whereas Leptin repressed the modification. In addition, SAHA or Leptin had no significant effects on the AcH4 acetylation binding with any regions of p21^WAF1/CIP1^ promoter. In MDA-MB-231 cells, SAHA alone or in combination with Leptin significantly increased acetylation levels of Ach3-k27, Ach3-k18 and Ach4-k5 residues. However, no clear change was found with Leptin alone at all. Overall, our data will inform future studies to elucidate the mechanisms of p21^WAF1/CIP1^ transcriptional regulation, and the functional roles of p21^WAF1/CIP1^ in breast cancer tumorigenesis.

## INTRODUCTION

Breast cancer has consistently been the top female malignancy worldwide. Approximately 70-80% of breast cancer patients are estrogen receptor-positive which expressed ER alpha (ER-α), and 10-20% are referred as triple-negative breast cancer (TNBC) which lacks to express ER, progesterone receptor (PR) and human epidermal growth factor receptor 2 (HER-2) [1, 2, 3]. Hence, identifying risk factors and screening specific transduction pathways involved in the process are important for understanding breast development and tumorigenesis.

Leptin, a 16 kDa peptide hormone of 146 amino acids secreted from white adipocytes, is located on chromosome seven (7q31.3) [4, 5]. Leptin exerts biological effects by binding to its corresponding receptors (Ob-Rs), and previous studies confirmed that Leptin is over-expressed in breast cancers [6]. Leptin activated ER expression in ER-positive breast cancer cell line MCF-7 via the corresponding signal transduction pathway and up-regulated expression of ER-dependent genes [7]. Some results demonstrated that in MDA-MB-231 breast cancer cells (a TNBC cell line), Leptin induced breast cancer cell proliferation by ERK1/2 and STAT3 phosphorylation, and can function on regulation of tumor-stromal interactions to enhance the breast cancer stem cell activity [8, 9]. Hence, Leptin may be an independent risk factor for breast cancer.

Although causes of breast cancer development and tumorigenesis are complex, they are related to abnormal gene expression and regulation. In fact, epigenetic changes are more common and significant in breast cancer tumorigenesis as these changes affect gene transcription without modifying the underlying DNA sequence, and can be stably inherited through cell division [10, 11]. In normal breast cells, histone acetyltransferase (HAT) transfer the hydrophobic acethyl group on acetyl coenzyme A to specific lysine residues on the amino terminus of chromatin core histones. This effectively neutralizes the amino group positive charge and increases the steric hindrance between DNA and histones, promoting DNA depolymerization and stretching [12, 13]. This also offers better binding between DNA template and transcription factors, regulatory factor complex, and RNA synthetase, and activates transcription of specific genes [14, 15]. Histone deacetylases (HDACs) remove acetyl groups of lysine amino-terminal residues from histones and restore positive charges, thereby integrating with negatively charged DNA to inhibit gene transcription [16, 17]. During breast cell dysfunction, HDAC activity is significantly enhanced, and disturbed gene expression equilibrium contributes to an imbalance between proliferation and apoptosis, thereby promoting breast tumorigenesis [18].

Suberoylanilide hydroxamic acid (SAHA), a second generation HDAC inhibitor, is used to treat continued deteriorating or recurrent T-cell lymphoma [19]. Previous work confirmed that SAHA has anti-tumor properties and that it could combine with HDAC catalytic sites [20]. This effectively inhibited HDAC activity, increased histone acetylation to perturb cancer cell transcription, induced cancer cell differentiation, arrested cell cycle progression, and promoted cancer cell apoptosis [21]. Other studies confirmed that SAHA inhibited expression of cyclin D1 and activated p21^WAF1/CIP1^ function, resulting in cancer cell cycle arrest and induction of cancer cell apoptosis [22, 23].

Here, we evaluated SAHA on Leptin-induced cell proliferation and invasion in the breast cancer cell line MCF-7 and MDA-MB-231. By measuring the changes in transcriptional regulatory regions of the p21^WAF1/CIP1^ promoter in the breast cancer cells, we observed the different effects of SAHA on regulation of p21^WAF1/CIP1^ expression in Leptin-induced breast cancer cells.

## RESULTS

### Real-time monitoring of Leptin-induced breast cancer cell proliferation

**Figure 1:**
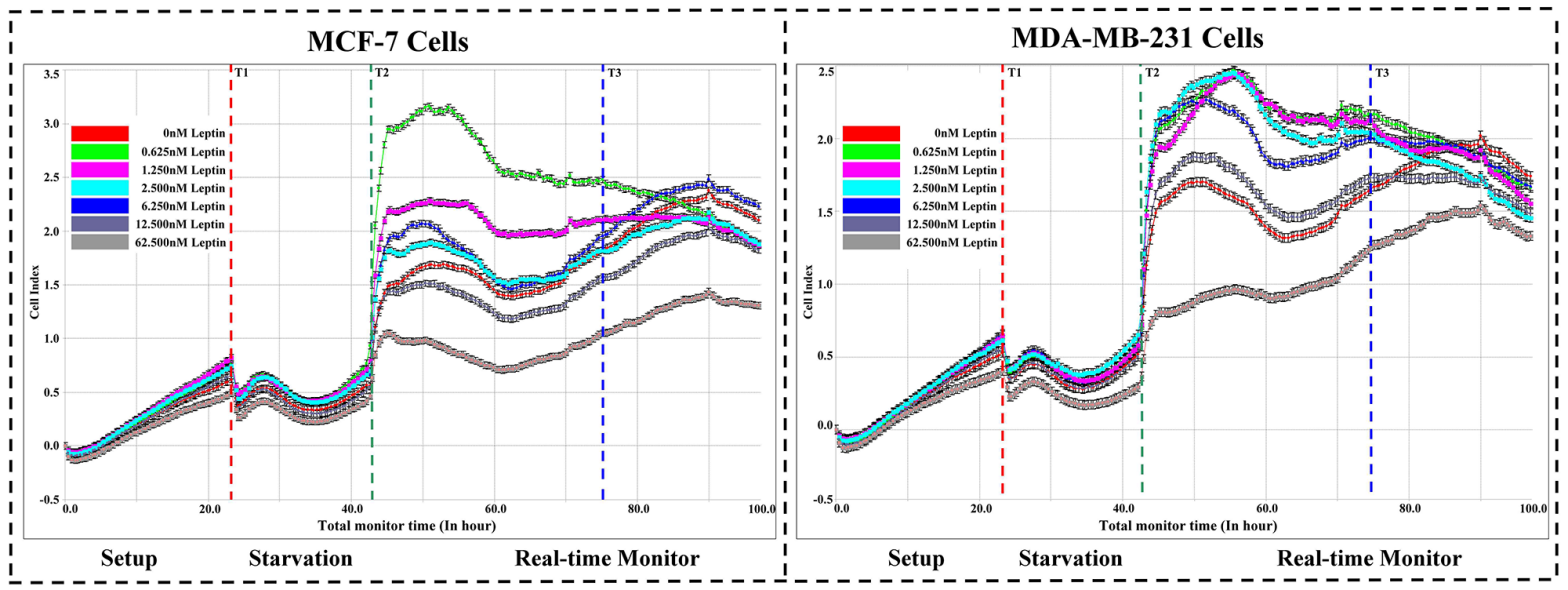
Real-time cell proliferation assays Breast cancer cell line MCF-7 or MDA-MB-231 was added into E-plate 16 in duplicates. After incubation with different concentrations of Leptin (0, 0.625, 1.25, 2.5, 6.25, 12.5, 62.5 nM), the cell kinetics were be monitored by xCELLigence Real-time Cell Analyzer (RTCA) DP system. For quantification, the cell index (CI) values at indicated time points were graphically represented at least three independent measurements. T1: Starvation; T2: Real time monitor; T3: Optimal treatment point.

We evaluated the effects of different Leptin concentrations on MCF-7 and MDA-MB-231 cell growth. Figure 1 indicates that 0.625nM Leptin tested in the experiments significantly induced MCF-7 and MDA-MB-231 cell proliferation. At the startup of Leptin treatment, the cell index curve changed irregularly, reflecting a stress reaction of the breast cancer cells. With the extension of exposure, the cell index curve trended to be stable and prolonged Leptin exposure up to 32 hours resulted in cell growth. Referring to our preliminary experiments, SAHA started to show anti-proliferative effect at 24 hours exposure [24]. Therefore, we determined the incubation of 0.625 nM Leptin for 32 hours exposure as the optimal combination treatment with SAHA. In addition, Figure 1 depicts that MCF-7 or MDA-MB-231 cells treated with higher Leptin concentrations (62.5 nM) had greater declines in cell indices curves, suggesting toxicity and cell growth inhibition. These data agreed with MTT assay data (data not shown).

### The effects of Leptin and SAHA on breast cancer cell viability and apoptosis

**Figure 2:**
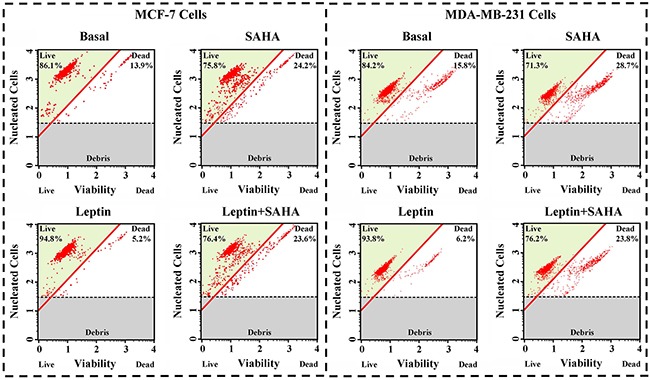
Cell Viability assay MCF-7 or MDA-MB-231 cell was plated into a 6-well plate. Then the cells were incubated in 0.625 nM Leptin or combining with 5 μM SAHA for 32 hours. Cell viability assay was performed by the Muse Count & Viability reagent and the results were obtained with Muse Count & Viability software module and the statistics were shown the percentage of viable cells.

Combining with real-time monitor data for Leptin and SAHA, we treated MCF-7 cells or MDA-MB-231 cells for 32 hours with 0.625 nM Leptin or 5 μM SAHA and measured viability and apoptosis for the breast cancer cells. For MCF-7 cells, compared with DMSO controls, SAHA treatment reduced cell viability, reducing them from 86.1% to 75.8% and dead cells increased from 13.9% to 24.2%. Leptin treatment increased viability, increasing living cells from 86.1% to 94.8%. For MDA-MB-231 cells, SAHA treatment also depressed the cell viability from 84.2% to 71.3%, and Leptin treatment induced the viability from 84.2% to 93.8% (Figure 2).

**Figure 3:**
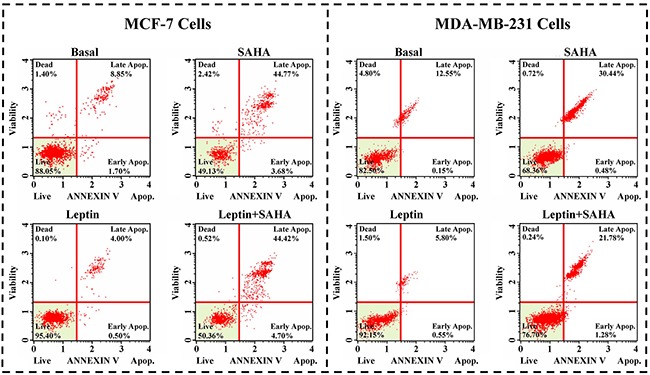
Cell apoptosis assay MCF-7 or MDA-MB-231 cell was incubated with Leptin and SAHA as described above, the cells were incubated with Muse Annexin V & Dead Cell reagent and the apoptosis array was determined by Muse Cell Analyzer. The statistics were shown the percentages of the cells represented by alive, apoptosis and dead population.

Compared with DMSO controls, SAHA treatment of MCF-7 cells significantly induced apoptosis (3.68% cells in the early stage and 44.77% cells in the later stage). With Leptin treatment, only 0.50% cells were in the early apoptotic stage and 4.00% cells were in the later stages. For MDA-MB-231 cells, SAHA alone repressed percentage of live cells from 82.50% to 68.36% and Leptin reversed this effect from 82.50% to 92.15% (Figure 3).

### The influences of Leptin and SAHA on breast cancer cell cycle

**Figure 4:**
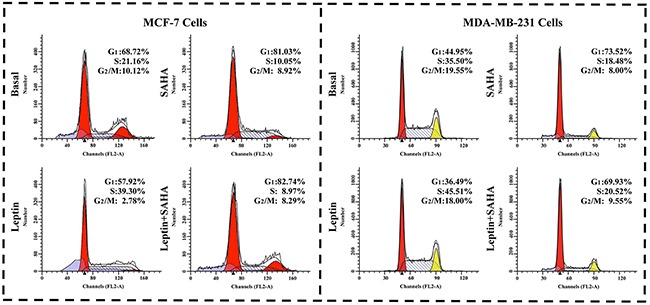
Cell cycle assay After treatment with Leptin or combining with SAHA, MCF-7 or MDA-MB-231 cell was stained with PI solution. The samples were analyzed on BD FACS Calibur flow cytometer was used to analyze the distribution of cell cycle. The experiment was repeated at least 3 times.

We measured the effects of Leptin and SAHA on the distribution of cell cycle. From the Figure 4, we found that SAHA treatment arrested most breast cancer cells at the G_0_/G_1_ phase. For MCF-7 cells, SAHA alone increased cells in G_0_/G_1_ phase from 68.72% to 81.03% compared to controls, and cells in the S phase decreased from 21.16% to 10.05%. SAHA treatment did not change cells in the G_2_/M phase significantly. With Leptin treatment, MCF-7 cells in the G_0_/G_1_ and G_2_/M phases decreased to 57.92% and 2.78% respectively; and cells in the S phase increased to 39.30%. For MDA-MB-231 cells, with SAHA treatment, the proportion of cells in G_0_/G_1_ phases was obviously increased from 44.95% to 73.52%, and that of the cells in S and G_2_/M phases was decreased from 35.50% to 18.48% and from 19.55% to 8.00% respectively as compared with control group. Leptin treatment resumed the cells in S phase up to 45.51% (Figure 4). Thus, Leptin and SAHA affect G_0_/G_1_-S-phase transition of the breast cancer cell cycle.

### The impact of Leptin and SAHA on the morphology of breast cancer cells

**Figure 5:**
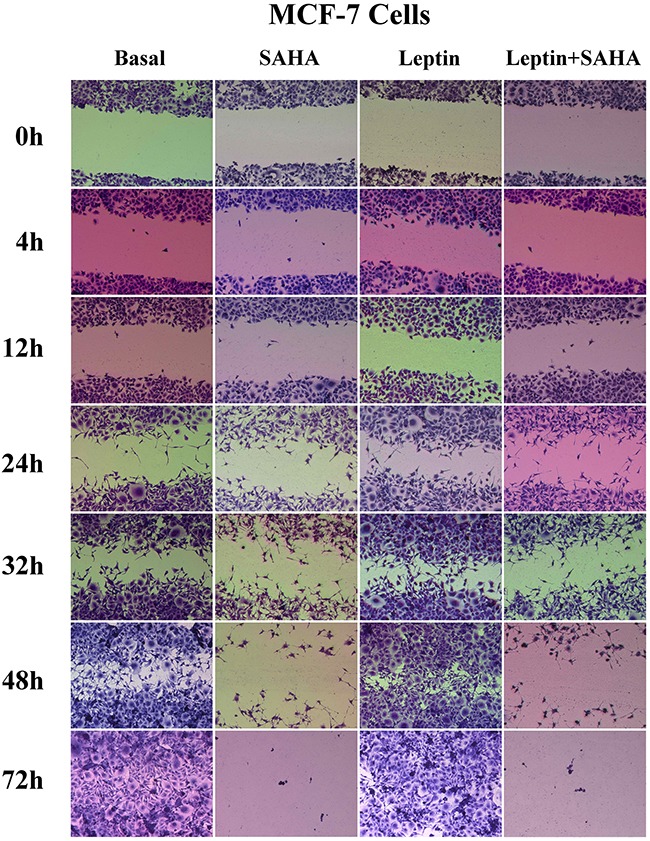
Wound healing assay MCF-7 or MDA-MB-231 cell was plated on a 12-well plate. After synchronization, the cell layer was scratched and incubated with Leptin or combining with SAHA for 0, 4, 12, 24, 32 and 48 hours respectively. The cells were stained and the images were captured by a Leica DMI6000 B microscope with phase contrast. The experiment was repeated at least 3 times.

**Figure 6:**
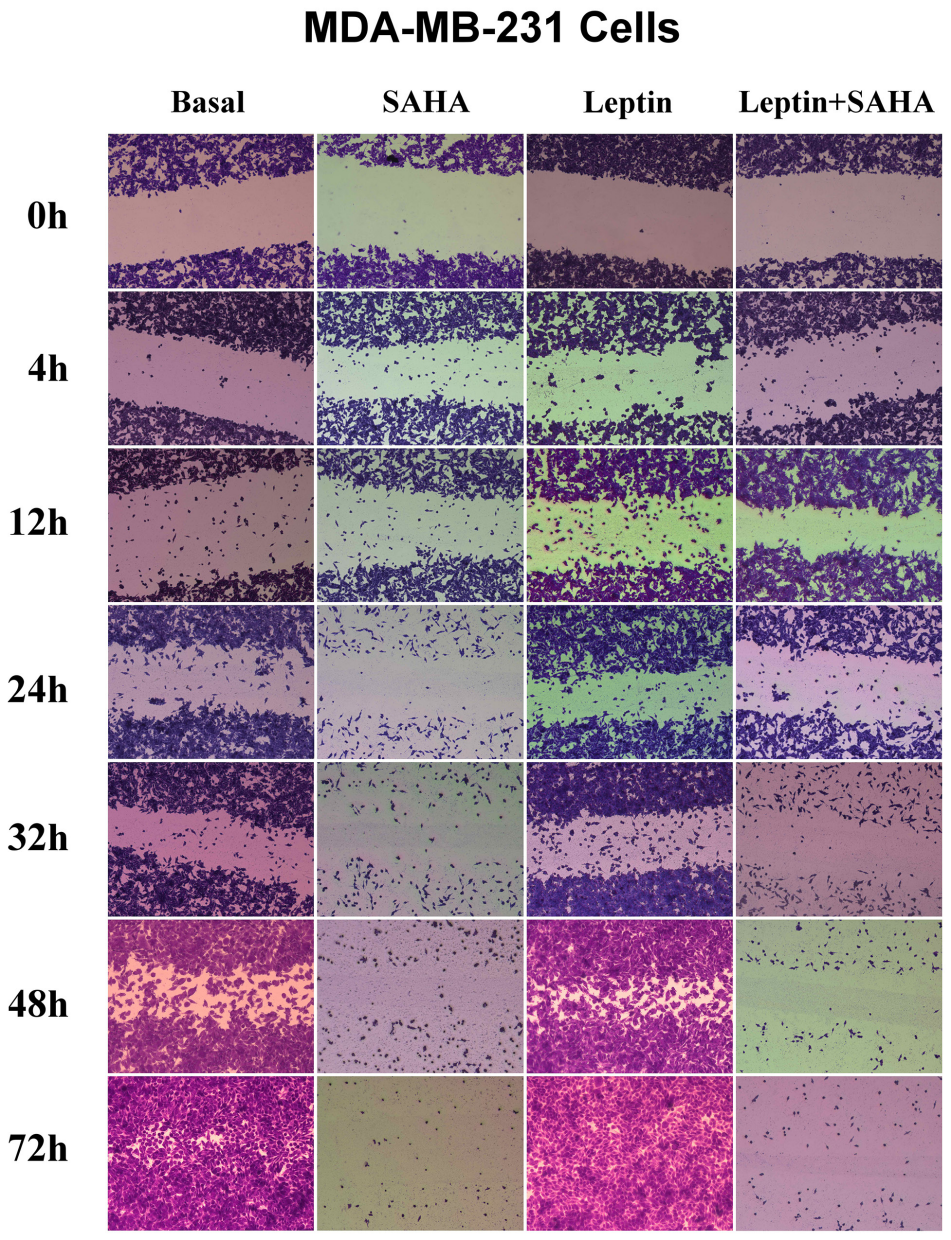
Wound healing assay MCF-7 or MDA-MB-231 cell was plated on a 12-well plate. After synchronization, the cell layer was scratched and incubated with Leptin or combining with SAHA for 0, 4, 12, 24, 32 and 48 hours respectively. The cells were stained and the images were captured by a Leica DMI6000 B microscope with phase contrast. The experiment was repeated at least 3 times.

To evaluate how Leptin and SAHA influence morphological features associated with breast cancer cell proliferation, we measured cell invasiveness by wound healing assay. It showed that 12 hours of Leptin incubation significantly induced MCF-7 cell migration toward the scratch center; and MCF-7 cells substantially filled scratch area gaps with 32 hours of Leptin incubation. After 48 hours of Leptin incubation, MCF-7 cells completely filled the scratch area. In contrast, longer treatment with SAHA significantly reduced the number of MCF-7 cells migrating toward the scratch center and surrounding area. When SAHA incubation time was up to 32 hours, most MCF-7 cells were dead and only few cells were alive scattered in the culture dishes (Figure 5). For MDA-MB-231 cells, 4 hours of Leptin treatment can initiate cell migration, and the cells obviously moved up to the central area with 24 hours treatment. After 48 hours of Leptin treatment, MDA-MB-231 cells mostly covered the scratch area. In contrast, 24 hours of treatment with SAHA significantly repressed the ability of cells migrating toward the scratch center (Figure 6).

### The inductions of Leptin and SAHA on cell cycle related molecules in breast cancer cells

**Figure 7:**
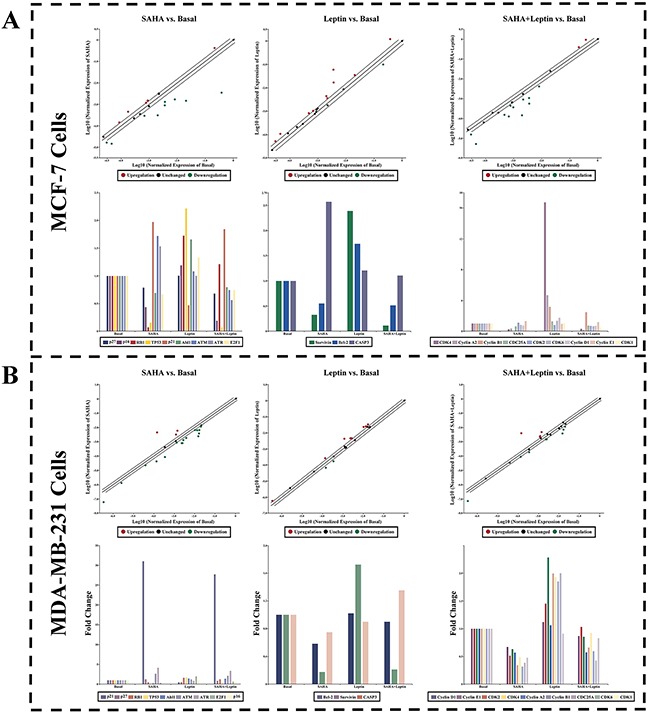
Real-time PCR Array Human real-time PCR arrays for cell cycle related gene were employed to validate the mRNA expression for MCF-7 or MDA-MB-231 cells treatment with Leptin and SAHA. Data normalization was based on correcting all *C*_t_ values for the average *C*_t_ values of GAPDH gene present on the array. Relative changes of gene expression in the array were calculated using the 2^^-ΔΔCt^ method. Three independent biological replicates were performed. A. MCF-7 cells; B. MDA-MB-231 cells.

In order to further clarify the impacts of Leptin and SAHA on breast cancer cell proliferation, we used cell cycle real-time PCR array to target mRNA changes of associated factors that can either positively or negatively control the transition of cell cycle. The results shown in Figure 7, SAHA significantly induced the expression of p21^WAF1/CIP1^ mRNA in MCF-7 and MDA-MB-231 breast cancer cells, and obviously inhibited Cyclin B1, p53, Cyclin A2 and RB1 mRNA expressions. On the contrary, Leptin reversed the effects completely. Besides, SAHA can stimulate the expressions of ATM, CDK1 and ATR in MCF-7 and MDA-MB-231 cells, but the effects were not clear in Leptin treatment alone.

### The affection of Leptin and SAHA on apoptosis related molecules in breast cancer cells

To characterize apoptotic roles and pathways of Leptin and SAHA in breast cancer cells, we used a proteome profiler antibody array to measure the changes in apoptosis-related protein expression in MCF-7 and MDA-MB-231 cells with Leptin and SAHA treatment.

**Figure 8:**
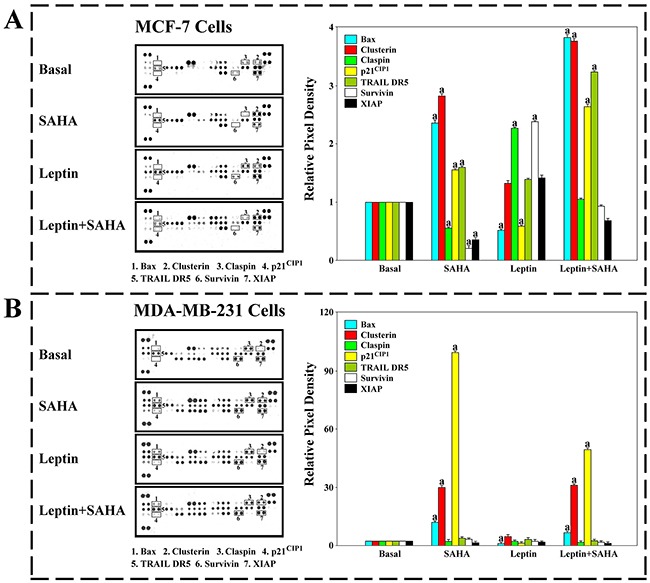
Human apoptosis antibody array The cells treatment with Leptin and SAHA were solubilized in lysis buffer. The samples were detected by apoptosis antibody array. Membrane intensity was acquired using chemiluminescence and pixel densities were measured using Gelpro Analyzer software. A. MCF-7 cells; B. MDA-MB-231 cells. (a) *p*<0.05 comparing with control (Basal).

For MCF-7 cells, SAHA treatment significantly induced expression of some pro-apoptotic factors, including Bax, and Clusterin, and inhibited Claspin, x-linked inhibitor of apoptosis protein (XIAP) and survivin protein expression while compared with controls. However, the effect was reversed by the treatment with 0.625nM Leptin. Also, the protein levels of tumor necrosis factor-related apoptosis-inducing ligand death receptor-5 (TRAIL DR5) and p21^WAF1/CIP1^, which are linked to apoptosis, were distinctly increased with SAHA treatment (Figure 8A).

For MDA-MB-231 cells with SAHA treatment, the protein levels of Bax, p21^WAF1/CIP1^ and Clusterin increased significantly, but the expression levels of claspin, XIAP and survivin maintained stable, and the levels of TRAIL DR5 were slightly increased. Leptin treatment alone showed the significant decrease in the levels of Bax and p21^WAF1/CIP1^ (Figure 8B).

### The functions of Leptin and SAHA on p21^WAF1/CIP1^ promoter related functional regions in breast cancer cells

**Figure 9:**
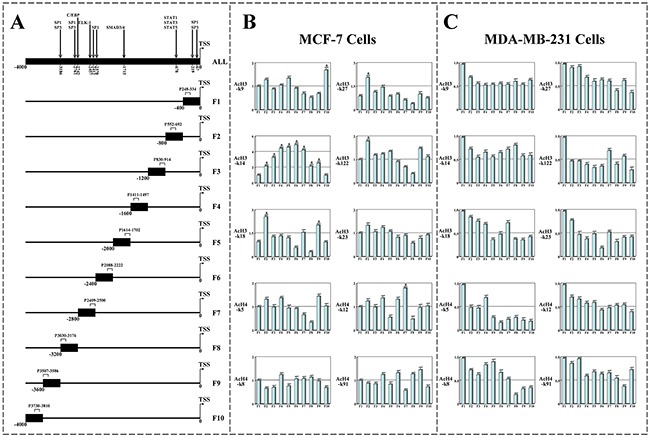
The screen of p21WAF1/CIP1 promoter regions binding with histone acetylated antibodies **A**. The 4kb sequences upstream of human p21^WAF1/CIP1^ promoter's TSS were obtained from online software and divided into 10 parts in average. To screen viral transcription motif of p21^WAF1/CIP1^ promoter binding with acetylated histone residues, a schematic depiction of human p21^WAF1/CIP1^ promoter amplified by real-time PCR was represented. Chromatin Immunoprecipitation (ChIP) Assays to display the affinity degrees of various AcH3 or AcH4 residues in p21^WAF1/CIP1^ promoter regions. **B**. DNA-ChIP assay for MCF-7 cells; **C**. DNA-ChIP assay for MDA-MB-231 cells. (a) *p*<0.05 comparing with control (Basal).

To characterize regulation of p21^WAF1/CIP1^ transcription in breast cancer cells with Leptin and SAHA treatment, DNA-ChIP arrays were performed and DNA was immunoprecipitated by anti-acetylated histone 3 (AcH3) or anti-acetylated histone 4 (AcH4) antibodies to different lysine acetylation residues. Real-time PCR was used to screen the high affinity region (-1~-4000) in the p21^WAF1/CIP1^ promoter region upstream of the TSS. A schematic map of the PCR amplification areas representing p21^WAF1/CIP1^ promoter region was drawn in Figure 9A.

For MCF-7 cells, the results were shown that the DNA binding domains of p21^WAF1/CIP1^ promoter with AcH3-k14 residues were broad and continuous, while only special p21^WAF1/CIP1^ promoter region (-400~-800) was bound with other histone acetylated residues (such as AcH3-k27, AcH3-k18 and AcH3-k122) in a non-continuous mode. However, p21^WAF1/CIP1^ promoter did not display an obvious binding characteristic with any AcH4 residues (Figure 9B). For MDA-MB-231 cells, whether histone AcH3 or AcH4 residues did not show much higher affinity in any p21^WAF1/CIP1^ promoter regions except in -1~-400 region (Figure 9C).

**Figure 10:**
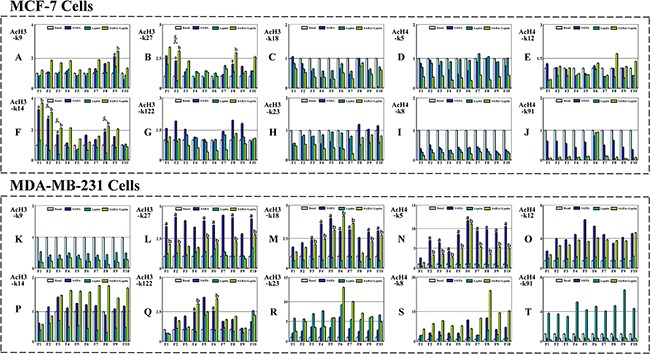
The functions of Leptin and SAHA on p21WAF1/CIP1 promoter functional region in breast cancer cells MCF-7 or MDA-MB-231 cell incubation with Leptin and SAHA was fixed and cell nuclei were digested with micrococcal nuclease. The cells were processed for Chromatin Immunoprecipitation assays according to the manufacturer's protocol. The anti-acetyl histone H3 or H4 antibodies were used for ChIP assays. Then DNA was purified and was run on an Applied Biosystems 7500 real-time PCR system. Final results were calculated using the 2^^-ΔΔCt^ method, using input Ct values instead of the GAPDH mRNA. (a) *p*<0.05 SAHA alone comparing with control (Basal); (b) *p*<0.05 The combination of Leptin and SAHA comparing with control (Basal); (c) *p*<0.05 Leptin alone comparing with control (Basal).

Meanwhile, we evaluated the changes of histone acetylation levels in breast cancer cells with Leptin and SAHA treatment. For MCF-7 cells, it showed that the acetylation levels of histone AcH3-k14 residues binding both in -400~-1200 and -2800~-3200 regions upstream of p21^WAF1/CIP1^ promoter were substantially elevated with SAHA treatment and significantly reduced with Leptin treatment (Figure 10F). Similarly, the acetylation levels of histone AcH3-k27 residues binding in -400~-800 region upstream of p21^WAF1/CIP1^ promoter were also different distinctly with Leptin and SAHA treatments (Figure 10B). However, no significant difference was found in the acetylation levels of any AcH4 residues binding with p21^WAF1/CIP1^ promoter with Leptin and SAHA treatments (Figure 10D, 10E, 10I, 10J). For MDA-MB-231 cells, the acetylation levels of AcH3-k27, AcH3-k18 and AcH4-k5 residues upstream of p21^WAF1/CIP1^ promoter were increased markedly with SAHA alone or combination with Leptin. However, no obvious change was found with Leptin treatment alone (Figure 10L, 10M, 10N).

## DISCUSSION

Breast cancer is one of the most common malignancies among women in the world, improving the chemoendocrine therapy and finding new ways to inhibit its proliferation and metastasis have important clinical significances [25, 26]. Leptin can serve as a growth factor to induce breast cancer cell proliferation and has been associated with severity of breast cancer stage and metastasis [27, 28, 29]. Here, we incubated breast cancer cells with exogenous Leptin and noted that low concentrations of Leptin (0.625 nM) significantly stimulated the cell growth, enhanced cell viability, minimized apoptosis, and increased breast cancer cells entering the S phase. Would healing assay indicated that Leptin stimulated cell invasion in both cell lines, implying that Leptin induced breast cancer cell growth through autocrine and paracrine pathways.

Cancer development and tumorigenesis have multiple causes, and epigenetic factors such as histone acetylation plays critical roles in gene transcription, DNA replication, and cell cycle progression [30]. In our assay, MCF-7 and MDA-MB-231 cells treated with 5μM SAHA were less viable and an increase in apoptosis was observed, suggested the inhibition of cell cycle progression. In addition, 32 hours incubation of SAHA caused significant cell death, inhibiting invasion and cell migration to peripheral areas. Furthermore, the cell cycle real-time PCR array data showed that Leptin and SAHA mainly influenced on the G_1_-S phase of cell cycle progression. SAHA apparently enhanced p21^WAF1/CIP1^ mRNA expression in both cell lines, and Leptin inhibited its expression. In this process, it was associated with the increase of p53, RB1 mRNA. Simultaneously, we found that there were different signaling pathways in response to the Leptin and SAHA treatment in the cell lines. For MCF-7 cells, the treatment of Leptin and SAHA focused on the impact of CDK4 and CyclinD1 complex functions, and for MDA -MB-231 cells, the role of Leptin and SAHA is reflected to affect CDK2 and Cyclin E complex expression. Nevertheless, the role of Leptin and SAHA is mainly to influence the transition of G_1_ to S phase.

Thus, we studied the expression of relevant apoptotic factors in the cells before and after Leptin and SAHA treatments. For MCF-7 cells, under the influence of Leptin, intracellular expression of Bax and Clusterin in the cells was significantly reduced but the expression of XIAP, survivin, and Claspin was elevated. Meanwhile, SAHA treatment had a reverse effect. For MDA-MB-231 cells with SAHA treatment, the protein levels of Bax, p21^WAF1/CIP1^ and Clusterin increased significantly, but the expression levels of claspin, XIAP and survivin maintained stable. Therefore, our data indicate that Leptin and SAHA may be related to the activation of apoptosis pathway in breast cancer cells, especially induced by the endogenous mitochondrial apoptosis pathway.

In the experiments, our works indicated that Leptin inhibited TRAIL DR5 expression, whereas SAHA significantly increased its product in MCF-7 cells. TRAIL DR5 is a functional receptor of TRAIL and responsible for delivering extracellular apoptotic signals and activating expression of intracellularly induced apoptotic proteins [31]. Thus, we speculated that the TRAIL DR5-mediated (or extrinsic) pathway may be involved in relevant biological effects in Leptin and SAHA treatment.

Finally, we measured p21^WAF1/CIP1^ transcription activity changes in breast cancer cells with Leptin and SAHA treatment. p21^WAF1/CIP1^, an important negative regulator of the cell cycle, inhibits cyclinD1-CDK4 and cyclinE-CDK2 expression to induce G_0_/G_1_ phase arrest [32, 33]. It also controls p53 protein function through negative feedback regulation, thereby depressed cell cycle and inducing apoptosis [34, 35]. Using DNA-ChIP, we evaluated the regulation of histone acetylation in the p21^WAF1/CIP1^ promoter region in response to Leptin and SAHA treatment.

Our DNA-ChIP data confirmed that the acetylated lysine residues had exhibited different binding activities in -1 to -4000 region upstream of p21^WAF1/CIP1^ promoter. The -400 to -3600 region of p21^WAF1/CIP1^ promoter showed a higher affinity with AcH3-k14 residues than other AcH3 or AcH4 residues at all. Using online biological software for bioinformatic analysis of p21^WAF1/CIP1^ promoter sequences from TSS to its upstream -4000 region, we noted that both regions from -1 to -800 and from -2300 to -3200 upstream of the p21^WAF1/CIP1^ promoter contained some regulatory elements for gene transcription including STAT1, SP1, SP3 and C/EBP. Combining with the data, we speculated that it maybe contains two important transcription regulatory regions in the p21^WAF1/CIP1^ promoter, one close to the proximal of the TSS and another located within more distal region from the TSS. p21^WAF1/CIP1^ promoter activity regulation within the proximal transcriptional regulatory regions was associated with the functions of STAT1, SP1, and SP3 regulatory elements, whereas p21^WAF1/CIP1^ promoter activity within the distal regulatory regions was mainly affected by SP1 and C/EBP. Our results were consistent with data reported elsewhere [36, 37], more verification of the relevant mechanism is necessary to confirm these data.

In addition, we identified the influence of Leptin and SAHA treatment on the acetylation levels of AcH3 and AcH4 residues binding with p21^WAF1/CIP1^ promoter in breast cancer cells. For MCF-7 cells, the acetylation levels of AcH3-k14 residues binding in both proximal and distal regions upstream of p21^WAF1/CIP1^ promoter were increased, and only the acetylation levels of AcH3-k27 residues binding with proximal regulatory regions upstream of p21^WAF1/CIP1^ promoter were changed. The histone acetylation levels of any AcH4 residues were not changed neither in proximal nor distal regions. However, for MDA-MB-231 cells, the histone acetylation levels of related AcH3 or AcH4 residues binding in p21^WAF1/CIP1^ promoter regions were enhanced distinctly with SAHA alone or combination with Leptin. Therefore, the data suggest that epigenetic modification such as histone acetylation is regulated in a manner specific to histone type, lysine residue and is limited to specific promoter regions in selective cell-type. Transcription activity at the p21^WAF1/CIP1^ promoter is altered due to the interactions between specific regions of p21^WAF1/CIP1^ promoter and specific protein acetylation.

In summary, we evaluated the influences of Leptin and SAHA on cell viability, cell cycle, and apoptosis in breast cancer cell lines, determined the mRNA and protein expressions of molecules associated with cell cycle and apoptosis, and identified the acetylated levels of AcH3 and AcH4 residues binding with p21^WAF1/CIP1^ promoter. Our data will inform future studies to elucidate the mechanisms of p21^WAF1/CIP1^ transcriptional regulation, and the functional roles of p21^WAF1/CIP1^ in breast cancer tumorigenesis.

## MATERIALS AND METHODS

### Reagents

Human MCF-7 and MDA-MB-231 cells were from American Type Culture Collection (ATCC) (Manassas, VA). RPMI-1640 medium, Leibovitz's L-15 medium, Fetal Bovine Serum (FBS) and Penicillin-streptomycin Cocktails were obtained from Thermo Scientific (Rockford, IL). Recombinant Human Leptin and Suberanilohydroxamic acid (SAHA) were purchased from Sigma-Aldrich (St. Louis, MO). Muse Annexin & Dead Cell kit, Muse Count & Viability kit were from Millipore (Darmstadt, Germany). Human Apoptosis Antibody Array kit was purchased from R&D Systems (Minneapolis, MN). Rabbit anti-acetyl histone H3 antibody (AcH3-k9), rabbit anti-acetyl histone H3 antibody (AcH3-k14), rabbit anti-acetyl histone H3 antibody (AcH3-k18), rabbit anti-acetyl histone H3 antibody (AcH3-k27), rabbit anti-acetyl histone H3 antibody (AcH3-k122), rabbit anti-acetyl histone H3 antibody (AcH3-k23), rabbit anti-acetyl histone H4 antibody (AcH4-k5), rabbit anti-acetyl histone H4 antibody (AcH4-k8), rabbit anti-acetyl histone H4 antibody (AcH4-k12) and rabbit anti-acetyl histone H4 antibody (AcH4-k91) were purchased from Abcam Inc (Cambridge, MA). High Pure RNA Isolation kit and Transcriptor First Strand cDNA Synthesis kit were obtained from Roche Diagnostics GmbH (Mannheim, Germany). Exprofile Human Cell Cycle Related Gene real-time PCR Array kit was obtained from Genecopoeia (Rockville, MD). Power SYBR Green PCR Master mix, RIPA Cell Lysis buffer and BCA Protein Assay kit were from Life Technologies (Austin, TX). Protease Inhibitor, Propidium Iodide and other chemicals were purchased from Sigma-Aldrich (St. Louis, MO).

### Cell culture

For all experiments, triplicate wells, tubes and reactions were run for each treatment and trials repeated at least three times with different cell preparations. MCF-7 cells were maintained in RPMI-1640 medium, MDA-MB-231 cells were cultured with Leibovitz's L-15 medium. 15% fetal bovine serum (FBS), 100 U/ml penicillin and 100 μg/ml streptomycin were supplemented with the medium. For treatments, cells were seeded on uncoated flat-bottomed plastic plates (cell densities of 5.0×10^5^/well for 6-well plates, 1.0×10^4^/well for 96-well plates). In other sets of experiments, cells were cultured at a density of 2.0×10^7^/60 mm tissue culture dish. Semi-confluent cells were starved for 24 hours in basal medium (with DMSO) without FBS and treated with different compounds.

### Real-time cell proliferation assays

1.0×10^4^/well of MCF-7 cells or MDA-MB-231 cells were added into E-plate 16 in duplicates and cultured for 24 hours with complete culture medium containing FBS. xCELLigence Real-time Cell Analyzer (RTCA) DP system was used to monitor cell kinetics across microelectronic sensors integrated into the bottom. Then the cells were starved in medium supplemented without FBS for 20 hours. The medium containing Leptin (0, 0.625, 1.25, 2.5, 6.25, 12.5, 62.5 nM) was added into the plate. The assay was monitored every 60 minutes for 60 hours. For quantification, the cell index (CI) values at indicated time points were graphically represented at least three independent measurements.

### Cell viability, apoptosis and cell cycle assay

MCF-7 cells or MDA-MB-231 cells were plated in 6-well plates at a density of 5×10^5^ cells per well. After synchronization with 5 μM DMSO (basal medium) without FBS for 24 hours, the cells were incubated in complete culture medium containing 0.625 nM Leptin or combining with 5 μM SAHA for 32 hours.

Cell viability assay was performed by the Muse Count & Viability reagent (Millipore) following the manufacturer's protocols. Following trypsinization, 2×10^5^ of harvested cells (50 μl cell suspension) was added with 450 μl Count & Viability reagent (Millipore). The results were obtained with Muse Count & Viability software module and the statistics were shown the percentage of viable cells.

For the apoptotic assay, 1×10^6^ of cells were transferred in suspension to a new tube and incubated with 100 μl of Muse Annexin V & Dead Cell reagent (Millipore) for 20 minutes at room temperature. The apoptosis was determined by Muse Cell Analyzer (Millipore) and the statistics were shown the percentages of the cells represented by alive, apoptosis and dead population.

For cell proliferation analysis, the cells treated with Leptin and SAHA were rinsed with PBS and incubated with trypsin. The lifted cells and the cells in the media were collected by centrifugation (1000 g, 5 minutes). The cell pellets were washed and 10 μl PI was added to the cell suspension. Each sample was then gently mixed and incubated for 15 minutes in dark. After incubation, 200 μl PBS was added to each sample. Samples were filtered through 200-mesh filters and then analyzed on BD FACS Calibur flow cytometer. Cell cycle was analyzed using FlowJo software.

### Wound healing assay

Briefly, MCF-7 or MDA-MB-231 cells (5×10^5^ cells/well) were plated on a 12-well plate and incubated for 24 hours in RPMI-1640 or Leibovitz's L-15 medium respectively. After synchronization, the cell layer was scratched using a 200 µl sterile pipette tip and incubated in complete culture medium containing 0.625 nM Leptin or combining with 5 μM SAHA in a time interval of 0, 4, 12, 24, 32 and 48 hours. The cells were washed twice with PBS and added 400 µl cell stain solution (Cell Biolabs, San Diego, CA) to each well for 15 minutes. The cells were subsequently washed three times with deionized water and images were captured using a Leica DMI6000 B microscope with phase contrast.

### RNA extraction and real-time PCR array

Total RNA was extracted from cells using high pure RNA isolation kit with DNase I. RNA was measured with a Nanodrop 2000 machine (Thermo Scientific). A total of 1μg RNA was used to synthesize first strand cDNA. Real-time PCR reactions were performed on a 7500 Real-time PCR system following manufacturer's instructions. Data normalization was based on correcting all *C*_t_ values for the average *C*_t_ values of GAPDH gene present on the array. Three independent biological replicates were performed.

Human real-time PCR array for cell cycle was employed to describe the cell cycle related mRNA expression. Relative changes of gene expression in the array were calculated using the 2^^-ΔΔCt^ (threshold cycle) method.

### Human apoptosis antibody array

The experiment was carried out in accordance with manufacturer's instructions. First, approximately 1×10^7^ MCF-7 or MDA-MB-231 cells with Leptin and SAHA treatment used the same concentrations as described above were solubilized in lysis buffer and centrifuged at 14000 g for 5 minutes. Protein concentrations of the resulting lysates were measured using a BCA protein assay kit. Next, each of antibody-coated array membranes was placed into the provided dish and 200 μg of prepared cell lysates were added each well of the dish to incubate at 4°C with gentle shaking overnight. The membranes were washed with wash buffer and then incubated with 1.5 ml lyophilized biotinylated antibodies for 1 hour on a rocking platform shaker. The mixture of biotin-conjugated antibodies was removed and membranes were incubated with horse radish peroxidase-conjugated streptavidin for 30 minutes. After a final wash, membrane intensity was acquired using chemiluminescence and pixel densities can be analyzed using Gelpro Analyzer software (Media Cybernetics, Rockville, MD). Densities were measured as a percentage of the positive controls included on each membrane. After subtracting background signals and normalization to positive controls, comparison of signal intensities between and among array images can be used to determine relative differences in expression levels of each protein between groups.

### Establishment of the p21^WAF1/CIP1^ promoter motif module

The 4kb sequences upstream of human p21^WAF1/CIP1^ promoter's transcriptional start sites (TSSs) were obtained from Transcriptional Regulatory Element Database of Michael Zhang Lab, Cold Spring Harbor Laboratory ( https://cb.utdallas.edu/cgi-bin/TRED/tred.cgi?process=searchPromForm). Moreover, all the sequences used in the experiments were verified from NCBI BLAST online software ( http://blast.ncbi.nlm.nih.gov/Blast.cgi?PAGE_TYPE=BlastSearch&SPEC=OGP__9606__9558&LOC=blasthome).

To screen viral transcription motif of p21^WAF1/CIP1^ promoter for breast cancer cells treatment with Leptin and SAHA, we set up a promoter motif model that divided the whole 4kb sequences upstream of TSSs into 10 fragments, and used Primer3 online software (http://bioinfo.ut.ee/primer3-0.4.0/) to design the real-time PCR primers for each fragment. The primers used for screening promoter motif are summarized in Table 1.

**Table 1:** The primers used for screening promoter motif are summarized in the table

Fragment	Primer Orientation	Sequence
F1	Forward	5′-TCCTCCTGGAGAGTGCCAAC-3′
	Reverse	5′-TTGGTGCGCTGGACACATTT-3′
F2	Forward	5′-TTCCCGGAAGCATGTGACAA-3′
	Reverse	5′-GCACCTGGAGCACCTAGACACC-3′
F3	Forward	5′-CCCGTTTCCCCAGCAGTGTA-3′
	Reverse	5′-GCCAGGAAGGGGAGGATTTG-3′
F4	Forward	5′-AGGCCAAGGGGGTCTGCTAC-3′
	Reverse	5′-CGGGGAGGACAGGCTTCTTT-3′
F5	Forward	5′-TGAAAGCAGAGGGGCTTCAA-3′
	Reverse	5′-ACCATCCAAAGGGCTGGTTG-3′
F6	Forward	5′-TGTCCTTGGGCTGCCTGTTT-3′
	Reverse	5′-AGCCCTGTCGCAAGGATCTG-3′
F7	Forward	5′-TTCTGCAGCCACCACTGAGC-3′
	Reverse	5′-GTGGAGCAGCATGGGGTAGG-3′
F8	Forward	5′-CCCACCTCAGCCACCTGAAT-3′
	Reverse	5′-GGGCAGATCACAGGGTCAGG-3′
F9	Forward	5′-AGTGGGCACATTTAGACATAGCAGGT-3′
	Reverse	5′-CCTCCCGGTCATGCCTTTC-3′
F10	Forward	5′-GTCAGGTGCCACTGGGGTCT-3′
	Reverse	5′-CGGTCCCCTGTTTCAATGCT-3′

### Chromatin immunoprecipitation (ChIP) assays

MCF-7 or MDA-MB-231 cells were set up into 100mm dish at a density of 6×10^6^ cells per well. After synchronization with basal medium, the cells were incubated in complete culture medium containing 0.625 nM Leptin or combining with 5 μM SAHA for 32 hours. Then the cells were fixed in 10ml 1% Formaldehyde (diluted in 1X sterile culture media) for 10 minutes at room temperature with rotation. 1X Glycine was added and incubated for an additional 5 minutes at room temperature to neutralize the Formaldehyde. The cell was washed with ice-cold PBS for two times and was detached by scraping in 1ml cold 1X PBS containing Halt Cocktail protease inhibitor (Thermo Scientific). The lysis buffer was used to break up and cell nuclei were digested with micrococcal nuclease (10 U/μl). The cells were processed for Chromatin Immunoprecipitation (ChIP) assays according to the manufacturer's protocol. The following antibodies were used for ChIP assays:rabbit anti-acetyl histone H3 antibody (AcH3-k9), rabbit anti-acetyl histone H3 antibody (AcH3-k14), rabbit anti-acetyl histone H3 antibody (AcH3-k18), rabbit anti-acetyl histone H3 antibody (AcH3-k27), rabbit anti-acetyl histone H3 antibody (AcH3-k122), rabbit anti-acetyl histone H3 antibody (AcH3-k23), rabbit anti-acetyl histone H4 antibody (AcH4-k5), rabbit anti-acetyl histone H4 antibody (AcH4-k8), rabbit anti-acetyl histone H4 antibody (AcH4-k12), rabbit anti-acetyl histone H4 antibody (AcH4-k91). Crosslinks were reversed on all chromatin samples by proteinase K digestion with 65°C incubation. Then DNA was purified using DNA clean-up columns following the manufacturer's instructions. The total input sample was diluted 1:10 (10% total input). For each sample and input DNA, 2.5 μl was used with Power SYBR Green PCR Master mix and was run on an Applied Biosystems 7500 real-time PCR system. The PCR conditions were 50°C for 2 minutes, 95°C for 2 minutes, followed by 40 cycles at 95°C for 15 seconds and 60°C for 1 minute. Final results were calculated using the ΔΔCt method, using input Ct values instead of the GAPDH mRNA. The basal sample was used as calibrator.

### Statistical analysis

*Student*'s *t*-test was used for data analysis. Data are presented as mean ± SEM. Values for *p*< 0.05 were considered statistically significant. The model included the main effects of treatments and replicates.
